# Integrins from extracellular vesicles as players in tumor microenvironment and metastasis

**DOI:** 10.1007/s10555-025-10287-x

**Published:** 2025-09-09

**Authors:** Corina Ciobanasu, Christophe Le Clainche

**Affiliations:** 1 Department of Exact and Natural Sciences, Institute of Interdisciplinary Research, Alexandru I. Cuza University, Boulevard Carol I, Nr. 11, Iasi, 700506 Romania; 2 Institute for Integrative Biology of the Cell (I2BC), Université Paris-Saclay, CEA, CNRS, Gif-Sur-Yvette, 91198 France

**Keywords:** Integrins, Extracellular vesicles, Tumor microenvironment, Cell migration, Exosomal integrins

## Abstract

Integrins constitute a large and diverse family of cell adhesion molecules that play essential roles in regulating tumor cell differentiation, migration, proliferation, and neovascularization. Tumor cell-derived exosomes, a subtype of extracellular vesicles, are enriched with integrins that reflect their cells of origin. These exosomal integrins can promote extracellular matrix remodeling, immune suppression, and vascular remodeling and are closely linked to tumor progression and metastasis, acting as pivotal players in mediating organ-specific metastasis. The present review aims to discuss recent insights into the role of integrins from extracellular vesicles in tumor cell initiation, proliferation, migration, and invasion. Beyond their functional roles in cancer progression, exosomal integrins hold relevant potential as diagnostic and prognostic biomarkers due to their tissue-specific expression patterns. They also represent promising therapeutic targets for disrupting tumor-stroma interactions and preventing metastatic spread. As research into exosomal integrins continues to expand, they are likely to provide valuable insights into cancer biology and innovative strategies in cancer diagnosis and treatment.

## Introduction

The integrin proteins consist of αβ heterodimeric transmembrane receptors, which can assemble 24 combinations of the 18 α and 8 β subunits, each with specific functions and binding preferences. Integrins are bidirectional signaling receptors and have an essential role in cell adhesion, migration, signaling, and communication with the extracellular matrix (ECM) and other cells [1, 2]. The αβ1-integrin heterodimers, for example, bind to ECM. The α subunit determines the specificity of the integrin for binding to certain ECM ligands, such as collagen, laminin, or fibronectin. Also, the αvβ-integrin family recognizes the short RGD motif (arginine–glycine–aspartic acid) found in ECM proteins like fibronectin or vitronectin, and is involved in diverse cellular processes, including cell adhesion, migration, and invasion. Additionally, αvβ-integrins can function as activators of transforming growth factor β (TGF-β) in tissues, thereby regulating various physiological and pathological processes, such as tissue remodeling, wound healing, and cancer progression [3].

The structure and function of integrins have been extensively documented in the literature, highlighting their critical role as transmembrane receptors that mediate ECM interactions, regulate intracellular signaling pathways, and contribute to various physiological and pathological processes [4, 5]. The functions of integrins are regulated through multiple mechanisms, including protein–protein interactions, conformational changes, and trafficking [6, 7]. In the majority of cell types, integrin functionality is contingent upon maintaining a finely tuned equilibrium between active and inactive receptor states on the cell surface.

**Fig. 1:**
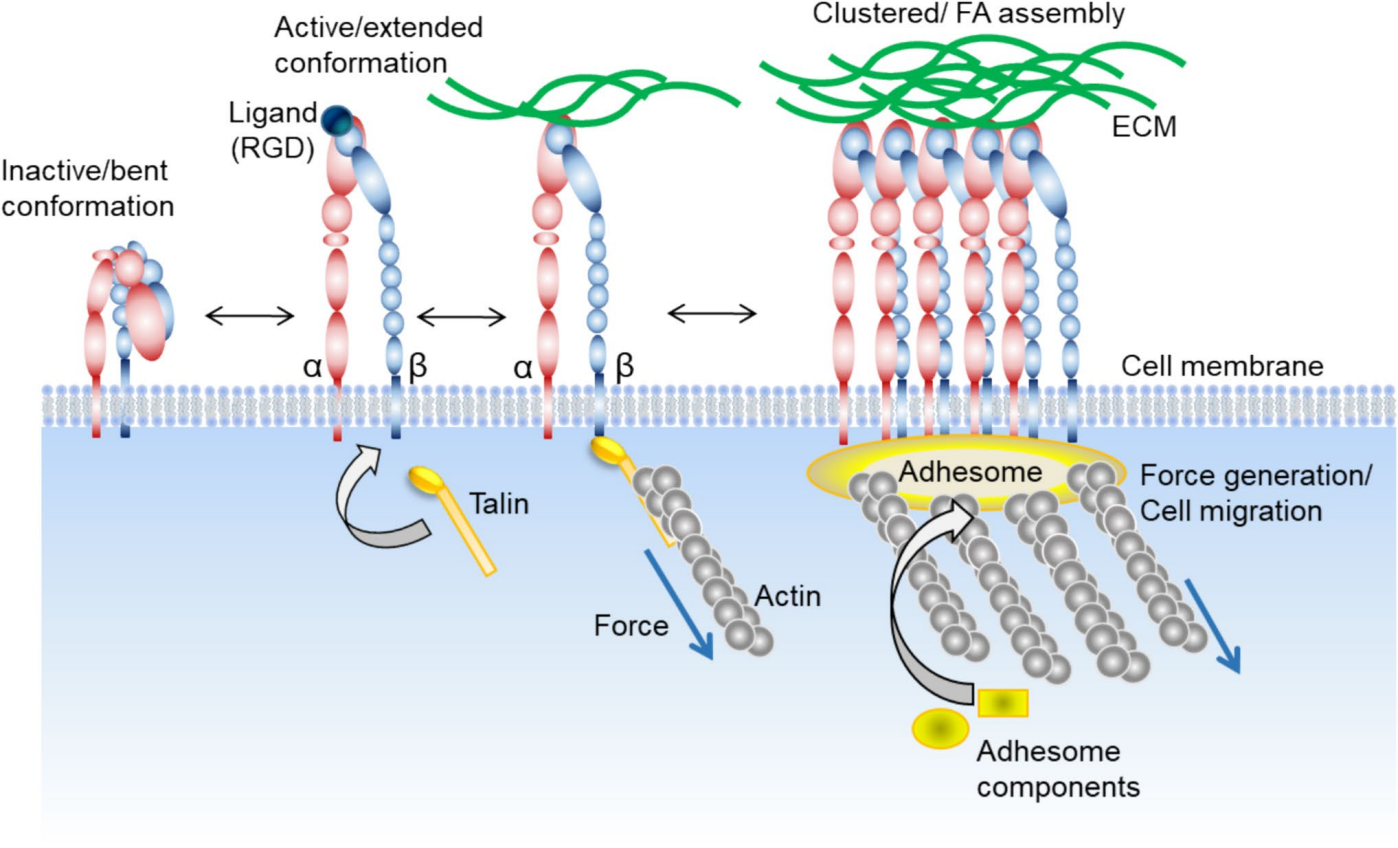
Function and activation of integrin proteins. In the inactive state, integrin adopts a closed conformation. Upon intracellular activation (“inside-out” signaling) by talin, integrin switches from its bent conformation to its extended conformation in which talin links integrin β-tail to the actin filaments making up the contractile actomyosin stress fibres. Additionally, the strong interaction between active integrins and ECM ligands triggers downstream (“outside-in”) signaling through multiple intracellular signaling pathways. After activation, integrin starts clustering so that nascent adhesions mature into larger focal adhesions in response to the tension applied by stress fibres

The cytoplasmic tails of integrins contain binding sites for cytoplasmic proteins known as integrin-associated proteins or integrin-binding proteins (such as talin and kindlin, for example). These proteins can interact with the cytoplasmic tails of integrins and modulate allosterically their conformation, thereby regulating the affinity of integrins for extracellular ligands. Also, integrins form dynamic multiprotein complexes known as focal adhesions (FA) at contact sites to the extracellular matrix [8]. The cytoplasmic tails of integrins play a critical role in the assembly and stabilization of these adhesion complexes by recruiting signaling and scaffolding proteins that facilitate the recruitment of actin filaments [9]. Integrin adhesions are highly dynamic structures, characterized by cycles of assembly and disassembly (see Fig. 1 for a schematic representation of actin activation). The lifetime and molecular composition of FAs exert a profound impact on extracellular matrix (ECM)-driven signaling pathways, thereby modulating cellular behavior.

Adhesion turnover is regulated by integrin endocytosis and exocytosis, which are often collectively referred to as integrin recycling. After endocytosis, integrins are recycled either through recycling endosomes, where they are returned to different regions of the membrane, or via early endosomes, where they are quickly returned to the same area of the membrane [10]. Endosomes containing internalized integrins and associated proteins serve as active sites for signaling [11]. Integrin endocytosis plays a role not only in adhesion turnover but also in transmitting “inside-in” signals. These signals are mediated through the recruitment of signaling molecules such as FAK (focal adhesion kinase) and other effectors, as well as by enhancing the signaling of co-trafficking of the growth factor receptors [12–14]. This dual functionality of endocytosed integrins underscores their importance in maintaining cellular communication and responses, particularly in dynamic processes such as cell migration and tissue remodeling.

Determining how the spatial and temporal distributions of integrins influence their recycling, endocytosis, and degradation and how they ultimately control key cellular processes such as adhesion, migration, and signal transduction in both normal and pathological contexts is of primary importance. Indeed, the study of integrin-containing extracellular vesicles holds great promise for advancing our understanding of cancer biology and is poised to provide novel perspectives and methodologies for precision oncology. By elucidating the role of exosomal integrins in tumor progression, metastasis, and the establishment of pre-metastatic niches (PMN), researchers can identify new biomarkers for early detection, prognostic evaluation, and therapeutic targeting. This line of investigation has the potential to revolutionize clinical prediction and pave the way for highly specific, integrin-targeted interventions that could improve treatment efficacy and minimize off-target effects in cancer patients.

## Extracellular vesicles

**Fig. 2:**
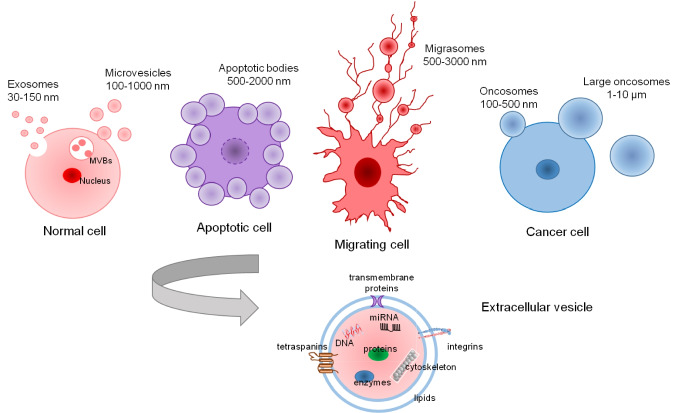
Classification and composition of extracellular vesicles (EVs). Schematic representation of different types of EVs according to their size: exosomes, the smallest population of EVs, generated by intracellular vesicle trafficking of early endosomes and multivesicular bodies (MVBs); microvesicles; migrasomes; apoptotic bodies; oncosomes; and large oncosomes

Extracellular vesicles (EVs) are lipid bilayer-delimited vesicles released by cells into the extracellular environment. They contain a variety of molecular cargoes, including nucleic acids (such as DNA, mRNA, and microRNAs), proteins, and lipids. EVs serve as carriers for intercellular communication, allowing cells to transfer information to neighboring or distant cells, as well as to the microenvironment [15, 16]. This transfer of bioactive molecules via EVs enables cells to influence various physiological and pathological processes, including cell signaling, immune response, tissue homeostasis, and disease progression. EVs are classified into different subtypes based on various criteria, including their size, biogenesis mechanisms, cargoes, and function (see Fig. 2). Generally, extracellular vesicles can be categorized as exosomes, microvesicles (also known as ectosomes or microparticles), and apoptotic bodies, as well as specialized subtypes such as migrasomes and oncosomes [17].

Exosomes are 30 to 150 nm in diameter and originate from the inward budding of the endosomal membrane, leading to the formation of multivesicular bodies (MVBs). Exosomes are delivered into the extracellular space once MVBs fuse with the plasma membrane. They usually contain proteins, lipids, RNA, and DNA, reflecting their endosomal origin [18]. Exosomes are frequent in cells like dendritic cells, B and T cells, mastocytes, and platelets but also in various biological fluids like cerebrospinal fluid, serum, synovial fluid, urine, tears, saliva, and breast milk [19]. Exosome activity is crucial for mediating various physiological and pathological processes, including cell communication, immune responses, cell signaling for regeneration (tissue repair and wound healing) and differentiation (stem cell differentiation, developmental processes) [18]. Their nanoscale size, which allows them to cross biological barriers, makes exosomes promising candidates for the development of drug delivery systems [20]. Moreover, exosomes can be used as a non-invasive biomarker for different diseases, namely Alzheimer's and prion diseases, viral infections, and cancer (melanoma, glioblastoma, ovarian, prostate and colon cancers) [21–23].

Microvesicles (ectosomes) are another type of EVs, usually with a diameter of 100 to 1000 nm, formed by the direct outward budding and fission of the plasma membrane. Microvesicles are similar to exosomes but can also include cytoplasmic components due to their formation process. Unlike exosomes, these structures lack specific markers but can contain various cell surface receptors (integrins, selectins, etc.), cytoskeletal proteins, enzymes, signaling molecules, carbohydrates, lipids, and genetic material including mRNA and microRNAs (miRNAs), depending on the cell of origin [24]. The formation of microvesicles is influenced by signaling molecules such as calcium ions, which trigger cytoskeletal reorganization, and enzymes like scramblases and flippases, which alter membrane asymmetry [25]. Microvesicles have different functions in cellular processes, such as cell-to-cell communication, immune modulation, tissue regeneration, and repair, as well as pathological processes (cancer and cardiovascular and neurodegenerative diseases) [26].

Apoptotic bodies are larger vesicles, ranging from 500 to 2000 nm in diameter. They are released during programmed cell death (apoptosis) and form as the cell membrane blebs and fragments into smaller vesicles named apoptotic bodies or, more recently, apoptosomes [27]. Apoptotic bodies contain fragmented DNA, histones, cytoplasmic organelles, chromatin remnants, degraded proteins, and other cellular components [28]. Apoptosis is an active and highly regulated process that plays a crucial role in various physiological and pathophysiological conditions [29]. Apoptosis occurs in normal cell turnover, regular development and functioning of the immune system, embryonic development, hormone-dependent atrophy, and chemical-induced cell death [30]. Impaired regulation of apoptosis can result in the development of numerous diseases. Excessive apoptosis can result in degenerative diseases, like Alzheimer’s and Parkinson’s, while insufficient apoptosis can lead to uncontrolled cell proliferation, as seen in cancers [31]. Fine-tuning apoptosis holds promise for treating a wide range of diseases. For example, cardiac infarction, which is caused by the lack of Bcl-2 protein, leading to excessive death of cardiomyocytes, can be prevented by overexpression of Bcl-2 [32]. Nerve growth factor (NGF) is a pro-survival factor in neuronal cells that can inhibit cell apoptosis and pathological death of neurons. Increasing Bcl-2 expression can avoid neurodegenerative diseases [33]. Bcl-2, an anti-apoptotic protein that inhibits cell apoptosis and promotes cell proliferation, can be found in extracellular vesicles (especially exosomes) released from certain cancer cells [34]. BCL-2 as well as BCL-2 siRNAs can be actively loaded into naturally secreted EVs for therapeutic purposes [35].

The less-known apoptotic bodies need more thorough studies to understand their role as specific markers. Up to date, only phosphatidylserine (PS) which is exposed on the outer membrane of these structures, is considered a cancer marker of apoptotic bodies [36].

Oncosomes are large, membrane-bound extracellular vesicles (100 to 500 nm) derived from cancer cells and are distinct from smaller extracellular vesicles like exosomes, in both size and function [37]. Studies have shown the formation of larger vesicles with an amoeboid movement, known as large oncosomes (LOs) with a size ranging from 1 to 10 µm [38]. Oncosomes contain oncogenic molecules like proteins, RNA, or lipids that could be transferred to neighboring cells, promoting cancer progression. Oncosomes are enriched with proteins involved in metabolic processes, particularly those related to glucose, glutamine, and amino acid metabolism. Ectosomes, in contrast, contain proteins associated with the cytoskeleton, such as actin and tubulin, and glycolytic enzymes [39]. The distinct cargo composition of oncosomes and ectosomes suggests different roles in cellular communication and disease. Depending on the cargo, oncosomes contribute to tumor growth, angiogenesis, and metastasis or to the establishment of a pro-tumorigenic environment by modulating the immune system and inducing resistance to therapies [40]. The differences between oncosomes and LOs at the cargo level are not clarified yet, but for prostate cancer, it was shown that cytokeratin 18 (CK18) was one of the most abundant proteins in LOs [39]. In glioma-derived EVs from the cell line U251, several pro-angiogenic factors were identified, including the vascular endothelial growth factor (VEGF), the C-X-C chemokine receptor type 4 (CXCR4), the transforming growth factor b type 1 (TGF-b1), and matrix metalloproteinases (MMPs) [41]. Oncosomes are being studied as potential biomarkers for cancer detection and prognosis, as they are shed into the bloodstream and can be detected in liquid biopsies (e.g., blood samples). Also, microRNAs from oncosomes can be used as potential therapeutic agents for inhibition of tumor angiogenesis [42].

The isolation and study of extracellular vesicles require a combination of physical separation methods, characterization tools, and functional assays. The choice of method depends on the type of EV (exosome, microvesicle, and apoptotic body), the goal of the research (e.g., purification and functional analysis), and the available samples. Often, multiple methods are used to ensure purity, specificity, and reliable functional analysis [43]. Differential ultracentrifugation remains the most common method, while techniques like immunoaffinity capture and microfluidics are gaining importance due to their precision. Table 1 shows an overview of the methods used to isolate and characterize various EVs.

**Table 1:** Overview of the methods used to study and isolate EVs

Method	Description	Applicability	Advantages	Limitations	Ref.
Differential centrifugation	Sequential centrifugation at increasing speeds to separate EVs based on size and density	Exosomes, microvesicles, apoptotic bodies, migrasomes	Simple, widely used, scalable	Time-consuming, potential co-isolation of different EV types	[44]
Density gradient centrifugation	Uses a density gradient (e.g., sucrose and iodixanol) to separate EVs based on buoyant density	Exosomes, microvesicles, apoptotic bodies, migrasomes	High purity, separates EVs based on density differences	Labor-intensive, may require ultracentrifugation	[45, 46]
Ultracentrifugation	High-speed centrifugation to pellet EVs from biological fluids or cell culture supernatants	Exosomes, microvesicles, apoptotic bodies, migrasomes	Efficient concentration of EVs	Requires specialized equipment, potential EV damage	[47]
Size-exclusion chromatography (SEC)	Separates EVs based on size using a chromatographic column filled with porous beads	Exosomes, microvesicles,migrasomes	High resolution, gentle on EVs	Limited throughput, may require combination with other techniques	[48]
Affinity isolation	Uses specific antibodies or ligands to capture EVs based on surface markers (e.g., CD63 for exosomes)	Exosomes, microvesicles	High specificity, can target specific EV subpopulations	Requires known markers, potential loss of EV integrity	[49]
Precipitation-based methods	Precipitation reagents (e.g., polyethylene glycol, PEG) are added to aggregate and precipitate EVs	Exosomes, microvesicles	Simple and can be done without specialized equipment	Co-precipitation of non-vesicular particles like proteins and nucleic acids	[50]
Ultrafiltration	Separation of EVs based on size by filtering the sample through membranes with specific pore sizes	Exosomes, microvesicles, oncosomes	Fast, simple, and cost-effectiv	Potential clogging of filters, lower purity, and risk of damaging EVs due to shear forces	[51, 52]
Flow cytometry	Analyzes and sorts EVs based on size, granularity, and surface markers	Exosomes, microvesicles	High resolution, quantitative analysis, ability to sort EVs	Requires labeling, limited sensitivity for small EVs	[53]
Nanoparticle tracking analysis (NTA)	Measures the size and concentration of EVs by tracking their brownian motion	Exosomes, microvesicles,migrasomes	Provides size distribution and concentration information	Requires specialized equipment, limited sensitivity for small EVs	[54, 55]
Transmission electron microscopy (TEM)	Visualizes EVs with high resolution using electron microscopy	Exosomes, microvesicles, apoptotic bodies, migrasomes	High-resolution imaging, provides detailed morphology	Requires sample preparation, not appropriate for quantitative analysis	[56]
Western blotting	Detects specific EV proteins using antibodies after isolating EVs	Migrasomes, exosomes, microvesicles	Specific protein detection, widely used	Requires known markers, not quantitative	[57]
Mass spectrometry	Identifies and quantifies proteins and lipids in EVs	Exosomes, microvesicles,migrasomes	Comprehensive molecular profiling	Requires specialized equipment, complex data analysis	[58]
RNA sequencing	Analyzes RNA content of EVs	Exosomes, microvesicles,migrasomes	Detailed RNA profiling, can identify RNA species present	Requires RNA extraction, complex data analysis	[59]
Microfluidics	Utilizes micro-scale devices to manipulate and analyze EVs, often with high precision and small sample volumes	Exosomes, microvesicles, migrasomes	High precision, low sample volume, integration with other techniques	Requires specialized device fabrication and design expertise	[60, 61]

## Cell migration and migrasomes

Migrasomes, first described a decade ago, are extracellular vesicles associated with cell migration that contribute to intercellular communication and the regulation of cellular environments [62]. These organelles, first reported in 2015, represent a novel class of extracellular vesicles with unique characteristics that distinguish them from previously identified EV subtypes, such as exosomes and microvesicles [63]. When cells move, they leave behind filamentous structures called retraction fibers (RFs). The vesicles that form at the tips and intersections of these RFs are defined as migrasomes (see Fig. 2). Migrasomes are generated during cell migration and are found in various cell types, including immune cells, metastatic tumor cells, and other specialized functional cells. Their sizes typically range from 0.5 to 3 μm and they contain smaller internal vesicles about 50 nm in diameter. It has been shown that the formation of migrasomes relies on the coordinated action of molecules, such as integrins, tetraspanins (TSPAN), and cholesterol [64, 65]. The aggregation of integrins at the migrasome formation site and their adhesion to the ECM are initial steps in migrasome biogenesis. Integrins facilitate the formation of retraction fibers and initiate the localized signaling and mechanical processes necessary for migrasome formation [64]. Integrins are enriched in focal adhesions (FAs), but other focal adhesion Markers such as paxillin, vinculin, or zyxin were not found in migrasomes, suggesting that integrin-enriched areas from these vesicles were not focal adhesions. Comparative analysis on migrasomes and FAs showed that the two structures share a Limited number of enriched proteins, about 1.5% and 1.7% from total (see Table 2 for examples of such proteins) [66, 67]. Tetraspanins are other crucial promoters of migrasome formation due to their ability to organize membrane microdomains, facilitate integrin function, and drive vesicle budding and cargo sorting. Their role in activating key signaling pathways further supports their involvement in the dynamic process of migrasome biogenesis [65]. TSPAN are not typically considered core components of focal adhesions, but they do interact with focal adhesion molecules, influencing their dynamics by modulating the function and organization of integrins and other adhesion molecules such as vinculin and talin [68]. A recent study showed that migration duration and speed are key parameters for migrasome formation [69]. Additionally, some viruses, drugs, peptides, genes, and cytokines can influence the formation of migrasomes [70–72]. Despite all these observations, the mechanism of migrasome formation is not yet fully understood. The average migrasome Lifetime is approximately 400 min, but the mechanisms governing the cycle from formation to clearance have not yet been precisely described. Similarly, the mechanism of formation of vesicle structure within the migrasomes is not known [63]. Migrasomes have a broad range of roles in intercellular communication, cell migration, tissue homeostasis, embryonic development, immune responses, and cancer progression [73]. These vesicular cellular organelles contain specific protein markers (e.g., carboxypeptidase Q (CPQ), EGF domain-specific O-linked N-acetylglucosamine transferase (EOGT), bifunctional heparin sulfate N-deacetylase/N-sulfotransferase 1 (NDST1), and phosphatidylinositol glycan anchor biosynthesis, class K (PIGK)) that can be used to distinguish between migrasomes and EVs, while Tetraspanin-4 and integrin are also present in exosomes [74].

**Table 2:** Proteins specific to focal adhesions and shared by focal adhesions and migrasomes and their role in migration

Protein	Role in focal adhesions	Role in migrasomes	Role in cell migration	Ref.
**Integrins**	Mediate attachment between the cell and extracellular matrix (ECM)	Involved in the budding and release of migrasomes	Regulate cell adhesion, signaling, and migration	[64, 75]
**F-actin**	Forms the contractile actomyosin stress fibres that connects to focal adhesions and generate traction force to drive cell movement	Associated with retraction fibers and migrasomes, provide structural support	Polymerizes at the leading edge to form membrane protrusions like lamellipodia and filopodia during migration	[76]
**Tetraspanins** (CD9, CD81)	Regulate the organization of protein complexes in membrane microdomains	Involved in the formation and function of migrasomes	Modulate cell adhesion, migration, and signal transduction	[65, 77]
**Arp2/3 complex**	Provides anchoring points between the lamellipodial actin network and adhesion proteins but its role at focal adhesions is independent on actin filament branching involved in lamellipodia	Facilitates actin remodeling needed for migrasome generation	Nucleation of branched actin filaments at the leading edge to form persistent lamellipodia	[63, 78]
**Talin**	Activates integrins and links them to stress fibres	-	Allows initial cell spreading necessary for the formation of a lamellipodium	[79]
**Vinculin**	Binds talin in response to actomyosin force to reinforce actin anchoring to focal adhesion	-	Reduces migration persistence	[80]
**Focal adhesion kinase (FAK)**	Regulates signaling pathways that control adhesion dynamics	Involved in signaling pathways within migrasomes	Promotes cell migration through focal adhesion turnover and signaling	[81]
**Paxillin**	Scaffolds protein interactions within focal adhesions	-	Coordinates signaling pathways that regulate cytoskeletal reorganization	[82]
**α-Actinin**	Crosslinks actin filaments in stress fibres associated with focal adhesions	Role in supporting the structural framework of migrasomes, ensures stability of retraction fibers	Controls the cell's traction force by balancing the activity of myosins	[83]
**Myosin**	Generates traction force by pulling on the actin filaments of the stress fibres, which is essential for the maturation, stability and renewal of focal adhesions	-	Allows traction of the cell body of adherent mesenchymal cells	[77]
**Kindlin**	Cooperate with talin to activate and cluster integrins	Contributes to migrasome biogenesis	Enhances integrin-mediated adhesion and signaling during migration	[84]
**Vimentin**	The intermediate filaments of vimentin control the assembly of stress fibres	Supports retraction fibers and migrasome stability	Controls the directionality of the traction forces generated by migrating cells	[69]
**Annexin**	Role in membrane repair processes, stabilizing adhesion sites under mechanical stress, regulate calcium signaling	Still need to be clarified	Repairs membranes and maintains cellular integrity, coordinates cytoskeletal remodeling for motility	[77]
**αβ-Tubulin**	Dynamic regulation and turnover of focal adhesions	Provides the structural and transport mechanisms necessary for migrasome formation and maintenance	Crucial for directional movement, cytoskeletal remodeling, and force generation during cell migration	[76, 85]

Migrasomes have been identified in a wide range of tissues and fluids, including ischemic brain tissue, platelets, blood, urine, intestine, eye, placenta, microglia, neutrophils, macrophages, lung, kidney, endoderm, the yolk syncytial layer of zebrafish embryos, and the middle layer of the chorioallantoic membrane in chicken embryos. Despite their widespread presence, their exact roles in physiological and pathological processes in the human body remain largely undefined. Further studies are essential to uncover the functional significance and potential implications of migrasomes in health and disease [86].

## Integrins of extracellular vesicles in the tumor microenvironment

Extracellular vesicles induce changes in tissue microenvironments by interacting with receptor cells and transferring proteins, lipids, and RNA cargos to cells. EVs can interact with target cells through multiple mechanisms, including direct fusion with the plasma membrane and internalization into the cell by endocytosis, macropinocytosis, and phagocytosis or fusion with endosomal membranes after internalization, such as those of early endosomes, late endosomes, or lysosomes [87]. Exosomes are secreted by most cell types, including cancer cells, and their contents can vary significantly depending on the cell of origin and the physiological or pathological state of the cell. However, there are also common molecules found in exosomes such as nucleic acids (predominantly RNAs), proteins, and lipids (cholesterol, sphingomyelin, and phosphatidylserine) [88]. Integrins are key receptors found in EVs which actively participate in cancer progression. Different types of integrins carried by extracellular vesicles and their role in cancer are depicted in Table 3.

**Table 3:** Different types of integrins carried by extracellular vesicles and their role in cancer

Integrin	Extracellular vesicles	Cancer type	Role in cancer	Ref.
Integrin αvβ3	Exosomes, microvesicles, apoptotic bodies, migrasomes	Melanoma, glioblastoma, reast cancer, ovarian cancer	Promotes angiogenesis, invasion, and metastasis	[89]
Integrin αvβ5	Exosomes, microvesicles, apoptotic bodies	Pancreatic cancer, breast cancer, liver metastasis, glioblastoma	Facilitates metastatic niche formation, enhances tumor cell invasion	[90]
Integrin α6β1	Exosomes, microvesicles, apoptotic bodies, migrasomes	Breast cancer, prostate cancer, ovarian cancer	Involved in tumor cell migration, adhesion to ECM, and metastasis	[91]
Integrin α6β4	Exosomes, microvesicles, apoptotic bodies, migrasomes	Breast cancer, lung metastasis, pancreatic cancer, thyroid cancer	Associated with aggressive tumor behavior, promotes invasion and metastasis	[90]
Integrin α4β1	Exosomes, microvesicles, apoptotic bodies, migrasomes	Leukemia	Aids in adhesion to bone marrow niche, promotes survival and proliferation	[92]
Integrin αLβ2	Exosomes, microvesicles, apoptotic bodies	Lymphoma, leukemia	Enhances immune evasion, promotes cell survival and migration	[93, 94]
Integrin αMβ2	Exosomes, microvesicles, apoptotic bodies	Various cancers (inflammatory roles)	Modulates immune responses, aids in tumor-associated macrophage (TAM) functions	[95]
Integrin αIIbβ3	Exosomes, microvesicles, apoptotic bodies	Glioblastoma, ovarian cancer	Promotes tumor growth, angiogenesis, and platelet-tumor interactions	[96]
Integrin α3β1	Exosomes, microvesicles, apoptotic bodies	Lung cancer, breast cancer, ovarian cancer, prostate cancer	Facilitates tumor cell migration, invasion, and metastasis	[97]
Integrin α5β1	Exosomes, microvesicles, apoptotic bodies, migrasomes	Prostate cancer, colon cancer, ovarian cancer, pancreas cancer	Enhances cell adhesion, migration, and invasion, involved in metastatic spread	[98]
Integrin β1	Exosomes, microvesicles, apoptotic bodies, migrasomes	Various cancers (e.g., breast, prostate, and colon)	Promotes cell adhesion, migration, invasion, and resistance to apoptosis	[99]
Integrin β3	Exosomes, microvesicles, apoptotic bodies	Endothelial cells in tumors	Supports angiogenesis, tumor growth, and metastasis	[100]
Integrin β4	Exosomes, microvesicles	Prostate cancer, breast cancer, gastric cancer	Enhances cell adhesion, migration and invasion	[100, 101]
Integrin α5β6	Exosomes, microvesicles, migrasomes	Prostate carcinoma, colorectal cancer, liver cancer, endometrial cancer	Enhances tumor growth, invasion, metastasis, and angiogenesis	[102, 103]

Integrins, which are present on the surface of EVs, can interact with corresponding ligands on recipient cells, facilitating their uptake and intracellular signaling. Heparan sulfate proteoglycans (HSPGs), which are glycoproteins present on the cell surface, have an important role in the recognition of EVs carrying integrin β3 and facilitating dynamin-dependent endocytosis, which is initiated by FAK-dependent phosphorylation. This coordinated process ensures that EVs carrying integrin β3 are efficiently internalized by recipient cells, allowing their cargo to exert biological effects within the cell [104]. Additionally, the efficiency of EV internalization is strengthened by integrin activation by talin and by the coexpression of tetraspanins (CD81 or Tspan8) in complex with the integrin [105].

**Fig. 3:**
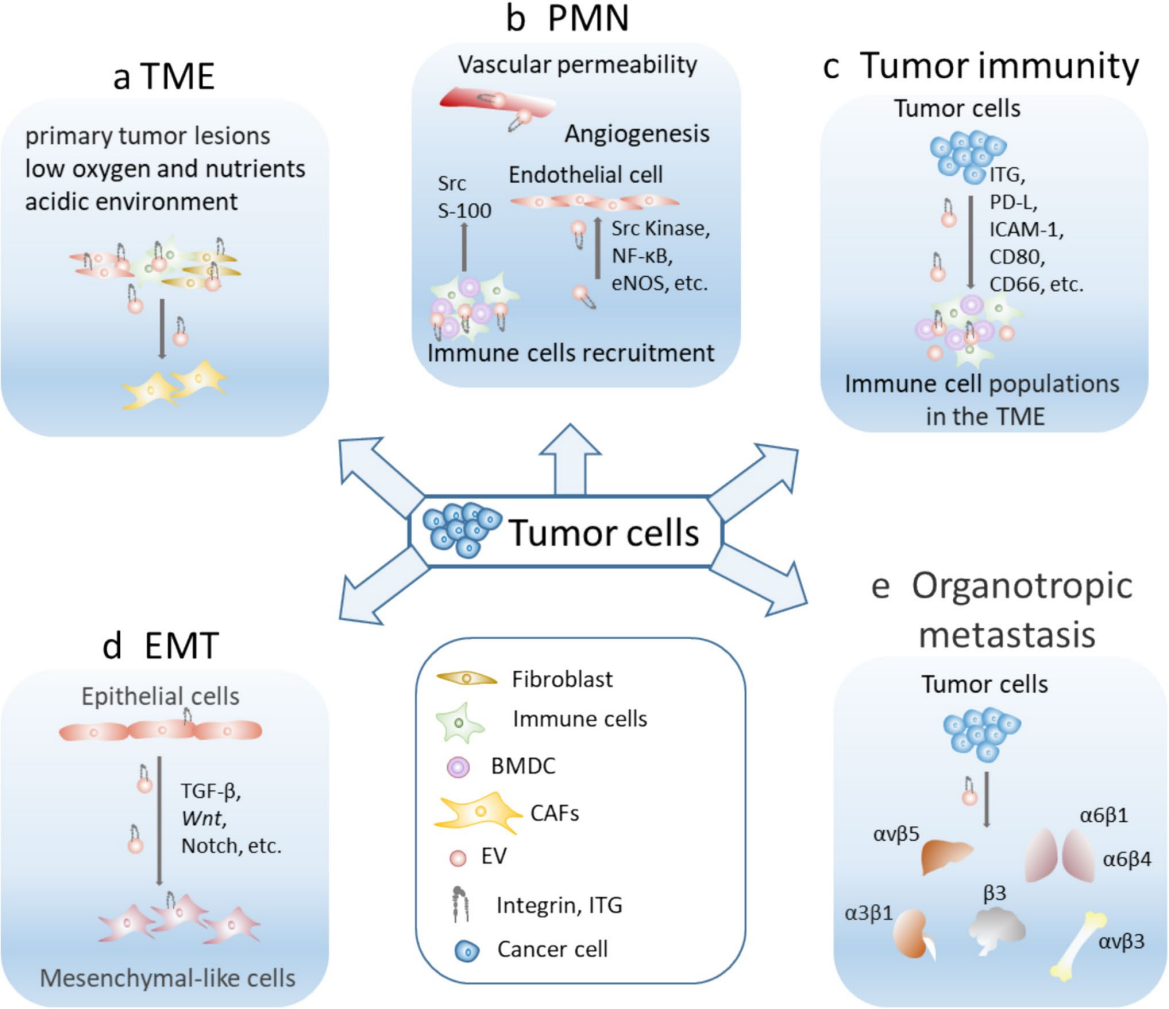
Role of EV/exosomal integrins in the TME, PMN, EMT formation, tumor immunity, and metastasis. **a** Exosomes carrying integrin (ITG), nucleic acids, lipids, etc., in the TME induce the differentiation of fibroblasts and promote tumor growth. **b** Tumor cells secrete signaling factors and specific integrins carried by EVs prior to metastasizing enabling them to establish PMNs by recruitment of multiple cell populations, including bone marrow-derived cells, immune suppressor cells, and fibroblasts. Enhanced blood vessel formation occurs through the stimulation of endothelial cell migration and proliferation. **c** Exosomal integrins modulate tumor immunity; immune escape is a defining characteristic of malignancies. **d** Exosomal integrins facilitate the transition of epithelial cells into mesenchymal-like cells by activating specific signaling pathways. **e** Exosomal integrins allow them to interact selectively with specific organs and initiate metastasis. TME, tumor microenvironment; PMN, pre-metastatic niche; EMT, epithelial-mesenchymal transition; CAFs, cancer-associated fibroblasts; BMDCs, bone marrow-derived cells; ECM, extracellular matrix

Although the function of EVs integrins in cancer progression is still under investigation, growing evidence showed that abnormal expression of integrins is involved in nearly every step (see Fig. 3), from tumor initiation to metastasis [106]. The expression of integrins αvβ3, αvβ5, αvβ6, α4β1, α5β1, and α6β4 in tumor cells is associated with the progression of lung, breast, pancreatic, prostate, and colorectal cancers [107].

The tumor microenvironment (TME) is a complex and dynamic network of various cellular components such as cancer-associated fibroblasts (CAFs), mesenchymal stromal cells and pericytes, and immune cells associated with vascular and lymphatic networks and embedded in ECM. These cells interact with cancer cells, creating a supportive ecosystem that regulates tumor initiation, growth, invasion, and metastasis [108]. The ECM within the TME provides structural support and acts as a reservoir for growth factors and signaling molecules, further modulating tumor behavior and conferring resistance to therapies [109]. Integrins have a dual function in cell migration, acting both as facilitators and regulators of the process. Exosomal integrins (*e.g.,* αvβ3, α6β4, and αvβ5) bind to ECM components such as fibronectin, collagen, and laminin, and these interactions create adhesion points between tumor cells and surrounding stromal or immune cells, enabling direct communication and structural anchorage [110]. During the early stages of tumorigenesis, integrin expression is downregulated, promoting tumor growth and dissemination. Subsequently, tumor cells enter the bloodstream, where integrin expression is upregulated, facilitating their adhesion to the vascular endothelium and inducing angiogenesis [111]. This enhances vascularization, providing nutrients and oxygen to tumor cells. Consequently, integrins serve as key adhesion molecules during the angiogenesis phase of tumor development. For example, exosomal integrins αvβ6 from prostate cancer cells can be transferred to endothelial cells that do not normally express epithelial-specific integrin αvβ6 and thus induce angiogenesis. The uptake of exosomal integrin αvβ6 is linked to an increase in pro-angiogenic survivin levels and a decrease in the angiogenesis-inhibiting phosphorylated STAT1 protein within endothelial cells [112].

Also, exosomal integrins stimulate recipient cells (e.g., fibroblasts) to release matrix metalloproteinases (MMPs), degrading ECM barriers. This remodeling enhances adhesion and migration pathways for tumor cells [113]. Tumor-derived exosomes carry integrins that preferentially bind specific ECM proteins in adjacent tissues. This receptive tissue microenvironment at a distance from the primary tumor is called the pre-metastatic niche (PMN). For instance, αvβ6 and αvβ3 integrins are linked with PMN formation in organs like the lungs. α6β4 integrins guide exosomes to laminin-rich environments (e.g., the brain), promoting adhesion to astrocytes or neurons [93]. Moreover, exosomal integrins adhere to immune cells, including macrophages and T cells, modulating their functions, orchestrating immune suppression and facilitating immune evasion. For example, adhesion via α4β1 or αvβ3 integrins can inhibit anti-tumor immune responses [114]. This facilitates recruitment and adhesion of tumor-associated macrophages (TAMs), which further remodel the TME to promote tumor growth. Integrin αvβ3 plays an important role in regulating interferon (IFN)-induced PD-L1 expression, a key mechanism by which tumors evade immune surveillance. Mechanistically, integrin αvβ3 facilitates IFN-mediated activation of the STAT1 signaling pathway, leading to increased transcription and expression of PD-L1 on the surface of tumor cells. This process suppresses the activity of cytotoxic T lymphocytes, thereby promoting immune escape. In a mouse model, silencing αvβ3 expression significantly impaired IFN-induced STAT1 phosphorylation, resulting in a marked reduction in PD-L1 expression. This downregulation of PD-L1 restored immune activity against the tumor, ultimately inhibiting tumor growth [115].

Recent studies showed an association between specific integrins from the surface of tumor exosomes and the site of future metastasis [116]. In some cases, EVs appear to play a key role in preparing a pre-metastatic niche and promote the survival and growth of metastatic cancer cells [117]. Exosomal integrins α6β4 and α6β1 bind to laminin-rich niches in lung tissue, activate Src and pro-inflammatory pathways in lung fibroblasts and endothelial cells, and create a PMN by increasing vascular permeability and recruiting immune cells [90]. Integrin αvβ5 binds specifically to fibronectin and vitronectin in hepatic sinusoids, triggers inflammatory signaling and ECM remodeling in liver stromal cells, and prepares a supportive microenvironment for metastatic colonization [90]. Exosomal integrins α6β4 and αvβ3 adhere to laminin in the blood–brain barrier, induce astrocyte activation via Src and NF-κB signaling, and promote brain metastasis by increasing vascular permeability and disrupting astrocyte integrity [118]. Integrin αvβ3 from exosomes interacts with osteoclast precursors, activating Src and FAK pathways, which promotes bone resorption by stimulating osteoclast differentiation and facilitates bone metastases by creating a favorable niche rich in growth factors [119].

Integrins are critical regulatory molecules on the surface of exosomes, playing a pivotal role in the early stages of metastasis by guiding metastatic cells to specific distant organs [90, 120]. Integrins from EVs can bind to their ligands on target cells facilitating EV uptake and subsequent biological effects. This organotropic behavior is mediated by the specific integrin expression profiles on exosomes, which allow them to interact selectively with target tissues, facilitating the formation of PMNs. These interactions that enhance the efficiency of metastatic colonization provide valuable insight into the mechanisms underlying tumor dissemination. Exosomal integrins which are derived from tumor cell integrins share similar tumor-promoting immune capabilities as their tumor cell counterparts. Chronic inflammation, a hallmark of cancer progression, is intricately linked to the functions of exosomal integrins, as they can exacerbate inflammatory responses that aid in remodeling the extracellular matrix, recruiting pro-tumor immune cells, and establishing PMNs. Consequently, integrin-guided preferential targeting of tumor-derived extracellular vesicles controls the specific organs where inflammation is triggered [95]. Exosomal integrins can target ECM proteins in distant organs and interact with recipient cells. Through interaction with these target cells, exosomal integrins can, for example, activate Src kinase signaling, a critical pathway that initiates downstream molecular events. This activation leads to the upregulation of the proinflammatory S-100 gene family, which plays a significant role in creating a PMN [90].

## Conclusions and future directions

Integrins are one of the largest families of cell surface receptors, expressed across nearly all cell types except erythrocytes. These transmembrane proteins facilitate the physical and functional coupling between the extracellular environment and the intracellular cytoskeleton. This connectivity enables integrins to regulate a wide array of cellular processes, including migration, proliferation, survival, and differentiation, contributing to diverse physiological and pathological processes, including wound healing, immune responses, and tumor progression. In this context, EV integrins and especially exosomal integrins have emerged as multifunctional mediators of cancer progression, playing distinct but interconnected roles in tumor biology. Firstly, through the horizontal transfer of integrin receptors via exosomes, cancer cells can acquire new functional capabilities that they would not normally exhibit, like invasive properties, enabling them to traverse tissue barriers and infiltrate new environments. This transfer not only increases the heterogeneity of the tumor cell population but also amplifies its metastatic potential. Secondly, the specific integrin expression profiles on exosomes have been shown to play a pivotal role in directing the formation of PMNs. By interacting with resident cells and the extracellular matrix in distant organs, exosomal integrins contribute to remodeling the microenvironment to favor tumor colonization. This organotropism is a key factor in the spread of cancer to specific secondary sites, such as the liver, lungs, brain, or bone.

Migrasomes, recently identified as a unique class of extracellular vesicles, are being increasingly recognized for their potential to significantly influence the tumor microenvironment. These vesicles, which form along retraction fibers during cell migration, carry a diverse array of bioactive molecules, including proteins, lipids, and RNA, that can modulate the behavior of surrounding cells and the extracellular matrix. By releasing their cargo into the TME, migrasomes can contribute to a range of processes, such as immune modulation, extracellular matrix remodeling, and the recruitment of stromal and immune cells. Moreover, migrasomes may promote tumor progression by enhancing the communication between cancer cells and other components of the TME, such as CAFs, endothelial cells, and immune suppressor cells. This interaction can lead to the establishment of a more favorable environment for tumor growth, angiogenesis, and metastasis. Migrasomes may also play a role in altering metabolic processes within the TME, thereby supporting the energy demands of rapidly proliferating tumor cells.

The use of EVs isolated from various body fluids, such as blood, urine, saliva, and cerebrospinal fluid, as sources for biomarkers in disease diagnosis, prognosis, and treatment guidance is rapidly approaching clinical application. EVs carry a rich cargo of biomolecules, including proteins, lipids, RNA, and DNA, reflective of their cell of origin. This unique feature makes them highly valuable for non-invasive liquid biopsies, enabling the detection and monitoring of diseases like cancer, neurodegenerative disorders, and cardiovascular conditions. As research continues to uncover the full potential of EV-based biomarkers, their integration into precision medicine approaches could revolutionize patient care by enabling earlier diagnosis, real-time monitoring, and personalized treatment strategies tailored to individual disease profiles. The ability of exosomal integrins to direct organ-specific metastasis highlights their potential as biomarkers for predicting metastatic patterns and as therapeutic targets for disrupting the metastatic cascade. As of now, the US Food and Drug Administration (FDA) has approved a total of seven drugs specifically targeting integrins [93].

These drugs have been developed for indications such as autoimmune diseases, where integrin inhibitors can disrupt the migration of immune cells into inflamed tissues, and cardiovascular disorders, where they help prevent platelet aggregation and thrombus formation. The development of such drugs represents a significant milestone in translating integrin biology into clinical practice. However, there remains substantial potential to expand the therapeutic applications of integrin inhibitors, particularly in oncology and metastatic disease management, where integrins play a pivotal role in tumor progression and organ-specific metastasis.

A comprehensive understanding of the roles and molecular mechanisms of exosomal integrins could open new ways for innovative clinical applications, including precision diagnostics and the development of integrin-targeted therapies, ultimately improving outcomes for cancer patients.

## Data Availability

No datasets were generated or analysed during the current study.
